# Clinical characterization of *Chlamydia psittaci* infections detected by targeted next generation sequencing in a Chinese tertiary hospital

**DOI:** 10.3389/fmed.2026.1744125

**Published:** 2026-01-26

**Authors:** Yu Song, Ruli Feng, Liying Sun, Mengjie Yan, Jing Zhou, Chenxue Qu, Lei Huang

**Affiliations:** Department of Clinical Laboratory, Peking University First Hospital, Beijing, China

**Keywords:** *Chlamydia psittaci*, diagnosis, pneumonia, psittacosis, targeted next generation sequencing (tNGS)

## Abstract

**Background:**

Psittacosis is a zoonotic disease caused by *Chlamydia psittaci* (*C. psittaci*). Its clinical symptoms are nonspecific, ranging from influenza-like symptoms to severe pneumonia. Thus, it is often ignored and underreported. To date, the report of *C. psittaci* infections detected by targeted next generation sequencing (tNGS) is still limited.

**Methods:**

tNGS was performed on the platform of Vision Medicals in patients with fever or respiratory infections from August 2024 to August 2025 in our hospital. The specimens included blood and bronchoalveolar lavage fluid (BALF). *C. psittaci* infection was confirmed by real-time PCR. Detailed clinical data of the included patients were collected and analyzed from electronic medical records.

**Results:**

Eight psittacosis patients were detected by tNGS and confirmed by real-time PCR. The median age of 8 patients was 69 years (range: 46–86 years). Five patients (62.5%) had a history of exposure to birds. Clinical symptoms included fever, cough, fatigue, headache and dyspnea. Patients exhibited normal or elevated white blood cell (WBC) counts with decreased lymphocyte counts. C-reactive protein (CRP), procalcitonin (PCT), and interleukin-6 (IL-6) levels were significantly elevated. The majority of patients (87.5%, 7/8) developed hepatic dysfunction. Pulmonary lesions were multi-lobar, presenting as consolidation, ground glass opacities and air bronchogram signs, with pleural effusion occurred in some cases. Following confirmation of *C. psittaci* infection, targeted antimicrobial therapies with tetracyclines (tigecycline or omacycline) were administered. All patients demonstrated significant reductions in inflammatory markers after treatments, with clinical symptoms improving until resolution. Follow-up chest computed tomography (CT) scans showed resolution of infection foci. All patients ultimately recovered and were discharged.

**Conclusion:**

tNGS is a promising tool for rapidly detecting *C. psittaci* infections. Early diagnosis of psittacosis with subsequently targeted therapies improved patients’ outcome.

## 1 Introduction

Community acquired pneumonia (CAP) could cause high morbidity and mortality. The death rate of pneumonia increases rapidly with age, from 0.317‰ among people of 65–74 years to 3.78‰ among those ≥ 85 years (1). Due to the complex diversity of pathogens in pneumonia, accurate and timely pathogenic diagnosis is difficult but vital for proper treatment and better prognosis (2). Due to the development of detection methods in recent years, such as metagenomic next generation sequencing (mNGS), the distribution of pneumonia pathogens has shifted in recent years, with an increase in some rare pathogens, such as *Chlamydia psittaci* (3, 4). *C. psittaci* is an obligate intracellular Gram-negative bacterium, which could infect birds, especially parrots, pigeons and mammals including humans. It can cause avian chlamydiosis in birds and psittacosis in humans (5). The clinical presentations of human psittacosis could vary from non-specific influenza-like illness to severe pneumonia. It could also combine with complications such as endocarditis, encephalitis, multiple organ failure and even death (6).

Pneumonia caused by *C. psittaci* is difficult to diagnose and often ignored by physicians due to its nonspecific symptoms and limited laboratory testing methods (7). Culture is the gold standard, but it is time-consuming and not appropriate for early diagnosis. Serological detection methods, e.g., immunofluorescence, take long detection time with unsatisfactory sensitivity and specificity. Molecular methods, e.g., real-time PCR, could only detect limited target pathogens in one assay, which is not suitable for detecting multiple rare pathogens simultaneously. mNGS has emerged as a powerful tool, which could simultaneously detect all microorganisms in a single test within 18–24 h, thus it is suitable to detect rare pathogens (3, 4).

Targeted next generation sequencing (tNGS), using targeted PCR amplification or probe capture techniques, enables simultaneous detecting hundreds of pathogens and antimicrobial resistance genes. mNGS could detect over 25.8 thousand kinds of pathogens within the database, and have the ability to detect emerging unknown pathogens (8). Compared with mNGS, tNGS could cover up to 95% clinical relevant pathogens (9). It is more cost effective, with higher specificity due to reduced human DNA interference (10). Thus, tNGS emerged as an important supplementary for mNGS recently. tNGS shortens the turnaround time (TAT) compared with mNGS or conventional methods. Cai et al. reported that TATs of tNGS, mNGS and blood culture were 16 h, 30.5 h and 50.1 ± 9.9 h, respectively (10). Notably, the reports of *C. psittaci* infections detected by tNGS were still limited (11). Thus the aim of this study was to describe the clinical characteristics of *Chlamydia psittaci* infections detected by tNGS in a Chinese tertiary hospital.

## 2 Materials and methods

### 2.1 Study design and data collection

This was a retrospective observational study approved by the ethical committee of Peking University First Hospital (no. 2025-1379). The inclusion criteria were patients with *C. psittaci* infections detected by tNGS in our hospital from August 2024 to August 2025. The detailed clinical data were collected from electronic medical records, including demographics, underlying diseases, epidemiological histories, comorbidities, treatments before and after diagnoses of psittacosis, outcomes, radiographic characteristics and laboratory findings. The exclusion criteria were as follows: (1) one or more above clinical data were missing or unavailable; (2) Real-time PCR detection of *C. psittaci* was negative. Eight patients following the inclusion and exclusion criteria were included in this study.

### 2.2 Real-time PCR for detection of *Chlamydia psittaci*

Specimens of the 8 patients in this study were sent to Beijing Center for Disease Prevention and Control (CDC) for confirmation following standard protocols. The MABSKY *Chlamydia psittaci* Detection Kit (Real-time PCR Method) was used. Ct value ≤34.5 was considered positive, and Ct value >37.5 was considered negative. When the Ct value was 34.5–37.5, the test should be repeated. The retested Ct value of 34.5–37.5 was judged as positive.

### 2.3 Sample preprocessing and nucleic acid extraction

The types of clinical specimens included blood and BALF. These samples were collected following standard aseptic procedure of our institution by experienced nurses, stored in sterile containers, and sent to Clinical Laboratory within 2 h. The standard operation protocols of Vision Medicals were followed. For blood, centrifugation was applied to separate cellular components. For BALF, purulent exudate and other sticky samples were pre-treated with trypsin. To minimize host DNA contamination of BALF, differential lysis strategies were employed.

DNA and RNA were extracted and purified using a magnetic bead-based method with nucleic acid extraction kits (VM020-50, Vision medicals) following manufacturer’s instruction. Quality control included quantification (Qubit dsDNA HS Assay), purity assessment (NanoDrop 260/280 and 260/230 ratios), and fragment size analysis (Bioanalyzer).

### 2.4 Library preparation, probe capture enrichment and sequencing

Total nucleic acid was extracted from clinical samples as described and subjected to library construction with the kit (VMRS0107-50, VMRS0108-50 Vision Medicals). Fragmentation was performed via enzymatic strategy to an average size of 200–300 bp. Mixed and fragmented DNA and cDNA underwent end repair, A-tailing, and adapter ligation. Purified library was amplified by P5/P7 primer pair and was purified again.

Targeted pathogen enrichment was achieved using a custom-designed probe panel (1.1 million probes, 120-mer length) targeting 486 pathogens, including bacteria, fungi, viruses, and parasites. Probes were synthesized with 3′ biotin and hybridized to fragmented nucleic acids with the kit (VM028-5, VMRS0056-16, VMRS0106-5, Vision Medicals). Hybridized complexes were captured via streptavidin magnetic beads, washed sequentially with low-salt and high-salt buffers to remove non-specific binders. Captured DNA was eluted in nuclease-free water and amplified via 12 cycles of PCR using indexed primers.

All libraries were pooled together after concentration determination. DNA nanoballs were made by using pooled library. Next generation sequencing was performed with Visionseq 1,000 (VM007-SE100S, Vision Medicals).

### 2.5 Bioinformatics analysis and report

Adaptor contamination, low-quality reads, duplicate reads and reads shorter than 40 bp were removed by Trimmomatic (v0.39). Low-complexity reads were removed by fastp (v0.23.4) default settings. The human sequence data were identified and eliminated utilizing Burrows-Wheeler Aligner software (BWA-0.7.17). Taxonomic classification of sequencing reads was performed using Kraken2 (v2.1.3) with default parameters. Kraken2 reference database was constructed using genomic sequences from RefSeq downloaded from NCBI RefSeq repository on 28 December 2024. Only alignments exhibiting a nucleotide identity of ≥ 96% to the reference genome were considered valid. To account for variations in sequencing depth across samples, we normalized the sequencing reads using reads per million (RPM). The optimal positive cut-off value for each species was determined by the parameter that yielded highest area under the curve.

tNGS results are reported following standard operation protocol from of Vision Medicals. There are a set of rules to adjudicate true pathogens versus contaminants. As *C. psittaci* is the rare pathogen, it is not easily contaminated from environment or reagent. All *C. psittaci* detected samples from this study was the only one detected within in the batch. The species specific reads were >3 and sequence identity matches were ≥96% of reference genomes in the database. The above criteria were used to adjudicate true pathogens versus contaminants. Two experienced staff with MD degree review the report independently.

## 3 Results

### 3.1 *Chlamydia psittaci* identification by tNGS and real-time PCR

From August 2024 to August 2025, tNGS was performed over 1,000 BALF and blood specimens from patients suspected of respiratory infections or bloodstream infections at Peking University First Hospital. These clinical specimens were collected for comprehensive pathogen detection using tNGS, aimed at diagnosing a broad spectrum of infectious agents rather than solely identifying *Chlamydia psittaci*. In our institution, tNGS is routinely prescribed by clinicians as part of the diagnostic workup. The procedure is available as a fee-based service, with patients being charged approximately 1,000 RMB per specimen in Beijing, China.

Among them, 8 patients were clinically diagnosed psittacosis by tNGS via identifying *C. psittaci* in BALF or blood samples. These samples were routinely sent to Beijing CDC for confirmation by real-time PCR. The results of real-time PCR targeting *C. psittaci* showed 100% agreement with tNGS. Written informed consents were obtained from the 8 patients.

### 3.2 Clinical characterization of 8 psittacosis patients

The demographic, clinical and imaging characteristics of all the patients were shown in Table 1. It included 5 males (62.5%) and 3 females (37.5%), with a median age of 69 years (range: 46–86 years). Five patients (62.5%) had a documented history of exposure to birds, among whom 3 patients had contact with pigeons, and 2 with parrots. The other 3 patients told no exposure history. *C. psittaci* could also be transmitted from other poultry besides bird exposure (11), and it could cause human-to-human transmissions via various pathways, including asymptomatic carriers (12). Thus the exposure history may be ignored or not realized by some patients. All patients exhibited fever, with the highest body temperature ranging from 38.6 °C to 40.4 °C. Six patients (75%) developed coughing. Three patients (37.5%) experienced fatigue and other symptoms included headache, shortness of breath, nausea and dyspnea. Five patients had underlying diseases: 4 had hypertension, 2 had heart diseases (coronary heart disease/atrial fibrillation), 2 had pulmonary diseases (chronic obstructive pulmonary disease/lung resection for cancer), and 1 had diabetes.

**Table 1:** Characteristics of 8 patients with *C. psittaci* infections.

Characteristics	Patient 1	Patient 2	Patient 3	Patient 4	Patient 5	Patient 6	Patient 7	Patient 8
Clinical manifestations								
Gender	Male	Male	Female	Male	Male	Male	Female	Female
Age	63	64	74	79	82	46	53	86
Specimen type	BALF	BALF/blood	Blood	BALF	BALF	Blood	BALF	Sputum
Temperature peak	38.7 °C	40.4 °C	39.0 °C	38.6 °C	39.0 °C	39.8 °C	40.0 °C	39.0 °C
Contact history	Keep pigeons at home	Keep parrots at home	Keep pigeons at home	Not described	Not described	Keep pigeons at home	Not described	Keep parrots at home
Clinical symptoms	Fever	Fever, cough, fatigue	Fever, cough, headache	Fever, cough, shortness of breath, fatigue	Fever, rigors, fatigue	Fever, cough	Fever, cough, nausea	Fever, cough, dyspnea
Underlying diseases	Hypertension, coronary heart disease	No	Lung resection for cancer	Hypertension	No	No	Hypertension	COPD, hypertension, diabetes, atrial fibrillation
Laboratory results								
tNGS reads	3,387	316,437/952	125	2081	57,295	63	21,089	2,320
WBC (3.5–9.5 × 10^9^/L)	5.49	8.04	6.88	14.60	13.50	11.35	9.40	8.08
Lymphocytes (1.1–3.2 × 10^9^/L)	0.89	0.31	0.63	0.61	0.24	0.53	0.61	0.66
CRP (0–8 mg/L)	81.29	271.10	173.66	263.92	211.27	225.56	206.36	86.40
PCT (<0.05 ng/mL)	0.12	21.35	0.31	2.26	5.30	1.25	1.17	0.44
IL-6 (<6.4 pg./mL)	70.64	—	—	118.86	104.44	156.12	133.76	—
ALT (9–50 IU/L)	25	91	54	34	55	27	69	11
AST (15–40 IU/L)	31	245	61	117	96	32	98	57
ALP (45–125 IU/L)	104	64	180	59	70	56	117	65
GGT (10–60 IU/L)	114	32	131	26	44	46	34	11
CREA (44–133 μmol/L)	88.50	160.10	77.40	81.70	94.10	79.60	85.00	91.40
eGFR (ml/min/1.73m^2^)	79.636	38.619	65.329	78.392	64.703	102.005	67.607	49.113
Imaging results								
Lesion site	Right lower lobe	Bilateral lower lobes	Left lung, right upper and middle lobes	Both lungs	Both lungs	Bilateral lower lobes, right upper lobe	Right upper and middle lobes	Left lower lobe, right middle and lower lobes
Imaging features	Consolidation	Consolidation, ground glass opacities, pleural effusion	Consolidation, ground glass opacities, pleural effusion, air bronchogram signs	Consolidation, ground glass opacities, pleural effusion, air bronchogram signs	Consolidation, ground glass opacities, pleural effusion	Consolidation, ground glass opacities, air bronchogram signs	Consolidation, ground glass opacities, pleural effusion, air bronchogram signs	Consolidation, ground glass opacities, pleural effusion

COPD: chronic obstructive pulmonary disease.

Most patients had white blood cell (WBC) counts within the normal range, while 3 patients (37.5%) had elevated WBC counts, and all patients exhibited decreased lymphocyte counts. All patients presented increased levels of C-reactive protein (CRP) and procalcitonin (PCT), and the 5 patients who underwent testing showed a significant increase in interleukin-6 (IL-6). All patients exhibited varying degrees of abnormal liver function, primarily manifested as elevated liver enzymes including aspartate transaminase (AST)/alanine aminotransferase (ALT)/alkaline phosphatase (ALP)/*γ*-glutamyl transpeptidase (GGT). One patient demonstrated increased serum creatinine and a significant decline in estimated glomerular filtration rate (eGFR), indicating renal impairment.

**Figure 1:**
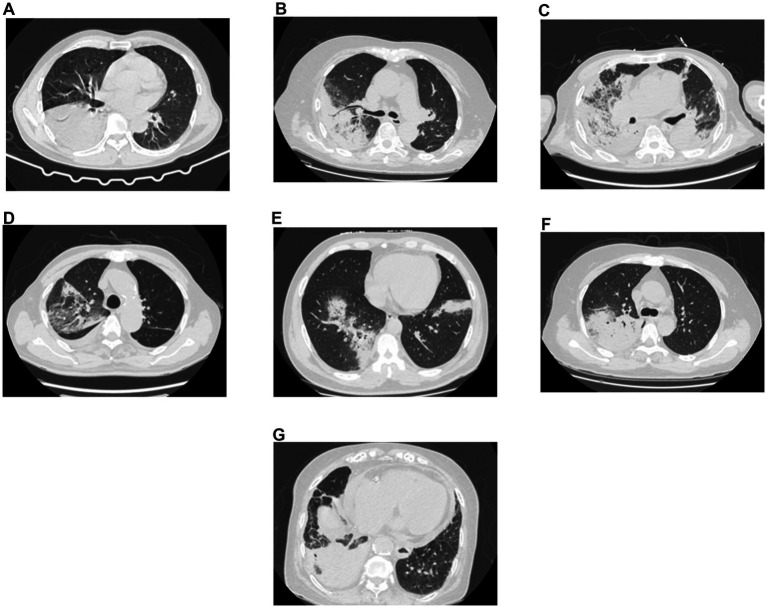
Image characteristics. **(A–G)** Represent patients 2–8, respectively. *C. psittaci* pneumonia can manifest as consolidation, ground glass opacities, air bronchogram signs, and pleural effusion.

Except for Patient 1 who underwent chest computed tomography (CT) at another hospital, the other patients were examined at our hospital. The imaging findings were presented in Table 1 and Figure 1. The majority of patients (87.5%, 7/8) exhibited multi-lobar pulmonary involvement, with only one patient (12.5%, 1/8) confined to a single lobe (right lower lobe). Six patients (75%) had bilateral lung involvement, while 2 patients (25%) had involvement limited to the right lung. All patients (100%) exhibited pulmonary consolidation, 7 patients (87.5%) showed extensively distributed ground glass opacities, 6 patients (75%) had pleural effusion, and 4 patients (50%) displayed air bronchogram signs. None presented with necrotic cavities.

### 3.3 Detailed diagnosis and treatment process for psittacosis patients

**Figure 2:**
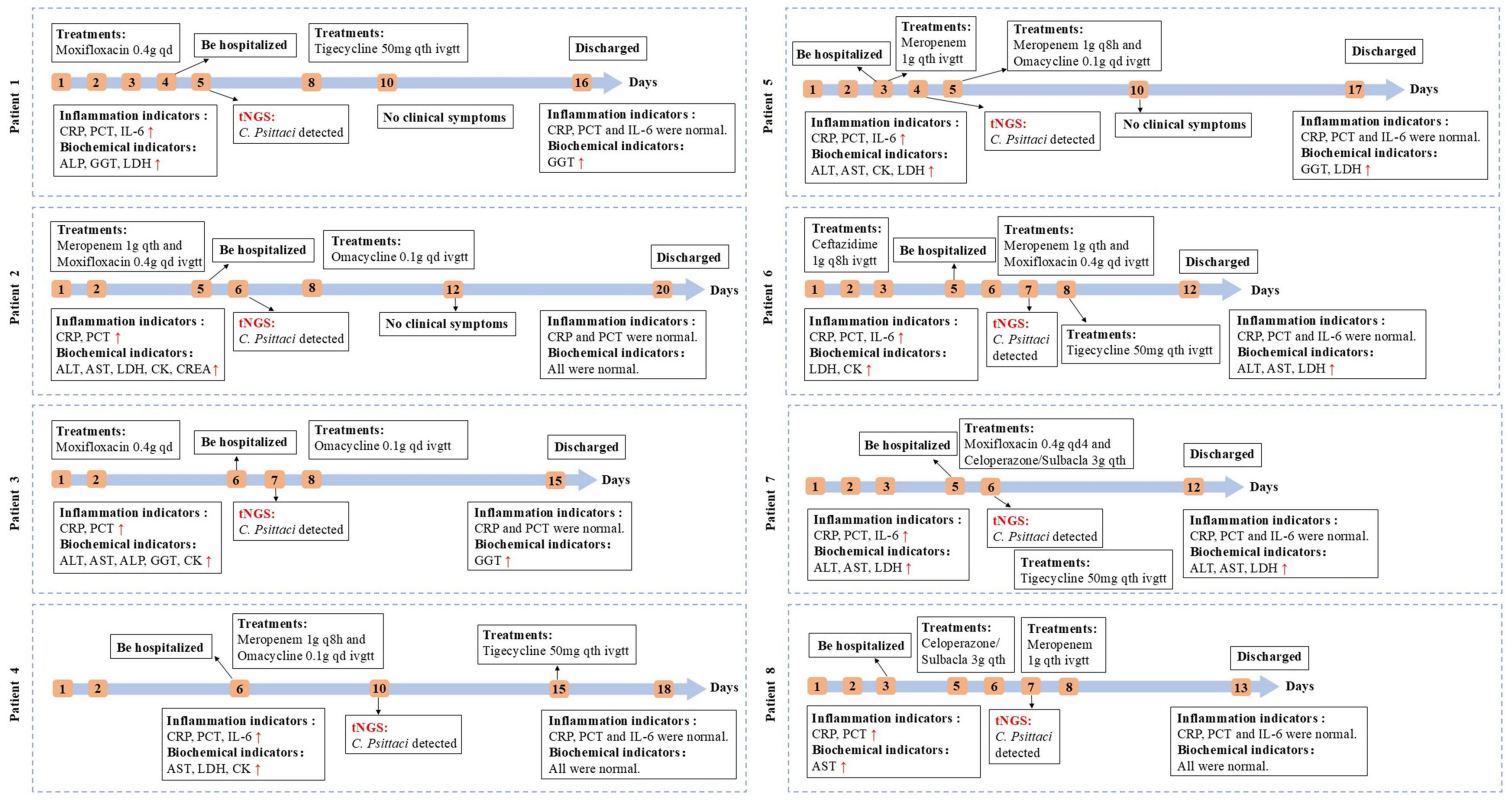
Treatment timeline of 8 patients. Treatment before and after diagnosis of psittacosis.

The diagnosis and treatment process for 8 patients was shown in Figure 2. The average time from symptom onset to hospitalization was 4.6 days (3–6 days), and the average time from hospitalization to diagnosis was 2 days (1–4 days). *C. psittaci* culture requires sophisticated cell culture or inoculation into yolk of embryonated chicken eggs. It is the gold standard, but is time-consuming and not appropriate for early diagnosis. Thus *C. psittaci* culture was not performed in our study, and subsequent antibiotic susceptibility results were not available. According to previous studies, tetracyclines (tigecycline and omacycline) are first-line agents for *C. psittaci* pneumonia (13). In severe and life-threatening cases, patients may require combined therapy with tetracyclines, macrolides and fluoroquinolones (14, 15). In this study, empirical antimicrobial therapies were initiated prior to confirmation. Following tNGS testing confirming *C. psittaci* infection, antibiotics were adjusted to tetracyclines (tigecycline or omacycline) for targeted antimicrobial treatment. The average length of hospitalization was 10.75 days (7–15 days). The average durations of empirical and targeted antimicrobial therapy were 4.62 days (2–7 days) and 8.00 days (5–12 days), respectively. After treatment, all patients demonstrated marked reductions in inflammatory markers, with clinical symptoms alleviating until resolution. Follow-up chest CT showed resolution of infection, and all patients ultimately recovered and were discharged.

## 4 Discussion

Psittacosis is a rare zoonotic disease (12). Due to the absence of typical clinical manifestations and imaging features, and the fact that routine respiratory infection screening typically does not include laboratory testing for *C. psittaci*, it is prone to being missed or misdiagnosed (16). Conventional antibiotics for CAP such as cephalosporins, penicillins and other *β*-lactam antibiotics are usually ineffective for psittacosis. Without prompt and definitive diagnosis, the condition may progress to sepsis and even multiple organs failure. A multicenter retrospective study conducted in China revealed a detection rate of 6.8% for *C. psittaci* infection among patients with severe community acquired pneumonia (SCAP) (16). There are probably two reasons for the high prevalence of psittacosis in China. Firstly, the psittacosis may be under-reported and under-diagnosis previously, however with the development of new techniques in recent years, more psittacosis patients have been detected by tNGS and mNGS. China has a large population, thus the number of patients detected with psittacosis has also been increasing in a short period of time. Secondly, with the living standards improving in recent years, more people have the habit of raising birds, especially pigeons and parrots, thus the exposure and risk factors increase. This indicated that *C. psittaci* had become an important emerging cause of SCAP, thus physicians should have enough awareness of psittacosis.

In recent years, the accuracy of psittacosis diagnosis has significantly improved with the development of various novel diagnostic tools. Advances in NGS technology have markedly enhanced the diagnosis and prognosis of such rare diseases. Currently, mNGS has become one of the primary methods for detecting *C. psittaci*. Previous study demonstrated that mNGS exhibited excellent performance in detecting *C. psittaci* (17). Based on statistical characteristics of zoonotic diseases over the past five years, the emergence of mNGS has significantly increased the detection rate of *C. psittaci*, particularly in China (5). It can detect all nucleic acid sequences in a sample through a single sequencing process, then identifying the pathogen while determining its taxonomic classification. Theoretically, a single test can accurately identify nearly all potential pathogens, including viruses, bacteria, fungi and parasites (18). mNGS showed superior performance in detecting *C. psittaci* with high sensitivity and specificity, and rapid turnaround time (13, 16, 19). Liang et al. showed mNGS could sensitively detect *C. psittaci* with a higher positive detection rate (100%, 13/13 *vs.* 46%, 6/13, *p* < 0.05) than conventional culture methods (19). However, the application of mNGS in lower respiratory tract infections still faces numerous challenges, such as complex operational procedures, difficult data analysis, and high testing costs (20). Additionally, mNGS is susceptible to interference from human host nucleic acid, which may compromise its diagnostic accuracy.

In order to solve the above problems, tNGS has technically compensated for some shortcomings of mNGS in pathogen detection. tNGS is a molecular detection method based on targeted amplification and high-throughput sequencing technology. Its detection sensitivity is seldom affected by human genome and background microbiome. In this study, tNGS was performed on the platform of Vision Medicals, targeting not only 482 pathogens (301 kinds of bacteria, 50 kinds of fungi, 110 kinds of viruses, and 21 kinds of parasites), but also 35 kinds of antibiotic resistance/virulence genes. Sample preprocessing is critical to minimize host DNA interference and enhance pathogen detection sensitivity. This technology also offers advantages such as low cost, minimal sample requirement, and easily standardized workflow. A prospective study conducted in China (21) demonstrated that tNGS achieved diagnostic performance comparable to mNGS in detecting pathogens causing lower respiratory tract infections, with the specific advantages in fungal detection. Given tNGS’s superior cost-effectiveness, it is recommended as the preferred method for pathogen detection in patients with lower respiratory tract infection in clinical settings.

However, there were only a few reports on the application of tNGS in detection of *C. psittaci*. This study analyzed the clinical characteristics of eight psittacosis patients identified through tNGS in a tertiary hospital. In this study, we evaluated the effectiveness of tNGS in early and rapid diagnosis of psittacosis, summarized diagnostic and therapeutic experiences, and assisted clinicians in better diagnosis.

Most patients in our study had a history of exposure to birds. The most common clinical symptoms included fever, cough, fatigue, headache and dyspnea. WBC counts were either normal or elevated. All patients exhibited significantly elevated levels of CRP, PCT, and IL-6. Patients exhibited varying degrees of liver enzyme abnormalities, suggesting possible liver involvement, which aligned with previous studies (13, 22). Since *C. psittaci* enters into the reticuloendothelial cells of liver and spleen after inhalation through respiratory tract, patients often exhibit abnormal liver enzymes despite primarily respiratory symptoms (23). One patient in this study exhibited renal impairment, suggesting the role of *C. psittaci* infection in affecting renal function, which also aligned with previous study (13).

The main CT manifestations were ground glass opacities and consolidation shadows, accompanied by air bronchogram signs. Some patients had pleural effusion. Interstitial involvement is a key distinguishing feature from other bacterial infections. The pathogen rapidly disseminates within alveolar space, leading to rapid imaging progression manifested as extensive consolidation. Consequently, CT images often reveal consolidation patterns resembling lobar or bulbar pneumonia, accompanied by well-defined peripheral ground glass opacities and reticular shadows. The coexistence of parenchymal and interstitial lesions constituted a relatively specific radiographic feature (24). Simultaneously, due to the relative absence of necrosis and cavitation within the consolidated areas, bronchi typically remain patent, thereby generating the air bronchogram signs.

Clinically, treatment for *C. psittaci* infection primarily targets patients presenting as pneumonia. Tetracyclines, macrolides, and fluoroquinolones could be used for treatments. Among these antibiotics, tetracyclines are first-line agents for *C. psittaci* pneumonia. In severe, life-threatening cases, patients may require combined therapy with tetracyclines, macrolides and fluoroquinolones (14, 15). Following confirmation of *C. psittaci* infection, patients in this study received targeted antimicrobial therapy with tigecycline or omacycline. All patients ultimately achieved favorable outcomes, attributable to timely diagnosis and treatment.

There are some limitations of this study. Firstly, only 8 psittacosis patients were included in this study, thus we could not perform further statistical comparisons (e.g., comparing different clinical manifestations or treatment options). Secondly, it was a retrospective study with small sample size, thus data bias could not be avoided. Further studies with larger sample size are still needed.

## 5 Conclusion

The clinical symptoms of psittacosis were nonspecific and varied, thus it was often ignored and underreported. tNGS is a promising and powerful tool in rapidly detecting *C. psittaci* and diagnosis of psittacosis.

## Data Availability

The original contributions presented in the study are publicly available. This data can be found in the repository of National Genomics Data Center of China (https://ngdc.cncb.ac.cn), with the accession number of PRJCA055726.
